# Heparin-Binding Protein in Bronchoalveolar Lavage Fluid as a Biomarker for Discriminating Severe Bacterial and Viral Pneumonia in Critically Ill Children

**DOI:** 10.1155/2023/6123911

**Published:** 2023-03-03

**Authors:** Caizhi Huang, Jie Zhang, Cong Zhang, Ping Zhang, Liya Mo

**Affiliations:** ^1^Department of Laboratory Medicine, Hunan Children's Hospital, Changsha, 410007 Hunan Province, China; ^2^Department of Laboratory Medicine, The Third Hospital of Yongzhou City, Yongzhou, 425000 Hunan Province, China

## Abstract

**Objective:**

This study is aimed at exploring the ability to use heparin-binding protein (HBP) in bronchoalveolar lavage fluid (BALF) to differentially diagnose bacterial infection from viral infection for severe community-acquired pneumonia (CAP) in critically ill children.

**Methods:**

A total of 181 children with severe CAP admitted to the intensive care unit (ICU) were included in this study. BALF and blood samples were collected within the first 24 hours of admission. BALF HBP and interleukin-6 (IL-6) concentrations and neutrophil percentage (N%) as well as blood HBP, IL-6, procalcitonin (PCT), C-reactive protein, white blood cell concentrations and N% were measured.

**Results:**

Of the enrolled children, 126 were confirmed to have bacterial pneumonia, and 55 were confirmed to have viral pneumonia. Blood HBP and PCT concentrations and N% and BALF HBP and IL-6 concentrations and N% were significantly higher in bacterial pneumonia than in viral pneumonia (*P* < 0.05). In the bacterial pneumonia group, HBP and IL-6 concentrations and N% in BALF samples were all significantly higher than those in blood samples (*P* < 0.001), and BALF HBP and IL-6 concentrations and N% were correlated with blood HBP and IL-6 concentrations and N%, respectively (*r* = 0.439, 0.250, and 0.235, *P* < 0.01). BALF N% and blood N% were both correlated with BALF HBP concentrations and blood HBP concentrations, respectively (*r* = 0.622 and 0.346, *P* < 0.001). ROC analysis revealed that BALF HBP showed the best ability to predict bacterial pneumonia, with an area under the curve of 0.994, a sensitivity of 95.24%, and a specificity of 100.00% at its optimal cutoff value of 74.05 ng/mL.

**Conclusion:**

BALF HBP might be a promising biomarker for the early discrimination of bacterial infection from viral infection in critically ill children with severe CAP.

## 1. Introduction

Community-acquired pneumonia (CAP) is an acute lung infection that results most commonly from viral or bacterial pathogens in children. Severe pneumonia requiring admission to the intensive care unit (ICU) is associated with a high mortality rate. The World Health Organization reported that more than 740,000 children under 5 years old died of pneumonia in 2019, accounting for 14% of all deaths in this age group. The diagnosis and treatment of CAP remain major challenges, mainly due to the practically difficult distinction between bacterial infection and viral infection according to clinical and radiological features [1]. This may lead to delayed antimicrobial therapy that is detrimental to disease progression and prognosis or to the administration of unnecessary and broad-spectrum antibiotics that promote the development of bacterial resistance. Currently, semiquantitative cultivation and identification of bacteria in respiratory samples remain the gold standard. However, culture is time consuming and can yield false-negative results due to previous antibiotic therapy or the presence of unculturable pathogens. Therefore, it is highly valuable to explore robust biomarkers to discriminate etiology and obtain a prompt indication of diagnosis in children with CAP.

Heparin-binding protein (HBP) is a cationic antimicrobial protein stored in the secretory and azurophilic granules of neutrophils and is prefabricated and rapidly released upon stimulation of leukocytic membrane-bound *β*_2_-integrins. When released from activated neutrophils, this multifunctional proinflammatory mediator activates immune cells, has broad-spectrum antimicrobial activity, and induces vascular leakage and edema formation [2]. Plasma HBP has been described as a good predictor of disease progression in children with severe CAP [3]. HBP has also been indicated as a biomarker for various bacterial infections in clinical studies. Increased concentrations of HBP in cerebrospinal fluid and urine have been associated with bacterial meningitis and urinary tract infection, respectively [4, 5]. Recently, HBP was considered a useful diagnostic marker in bronchoalveolar lavage fluid (BALF) for the detection of ventilator-induced lung injury in intubated patients and pulmonary infection in lung transplant recipients, in sputum for the determination of airway inflammation in children with cystic fibrosis, and in lower airway samples for the diagnosis of ventilator-associated pneumonia [6–9]. However, there are few data about the association between HBP concentrations in BALF and the etiology in children with CAP. In this exploratory study, we evaluated whether HBP in BALF from critically ill children with severe CAP can be used as a biomarker for discriminating pathogens.

## 2. Materials and Methods

### 2.1. Study Population

This retrospective observational study was based on data collected from July 2018 to December 2021. All children admitted to the ICU of Hunan Children's Hospital with severe CAP who underwent bronchoscopic bronchoalveolar lavage (BAL) within the first 24 hours of admission were enrolled. The definition of CAP was applied according to the 2011 guidelines of the British Thoracic Society for the management of CAP in children [10]. All children's information was anonymized and deidentified during the analysis. Informed consent that medical records might be collected and used for clinical research was obtained from the legal guardians of all children at admission in this study. This study protocol conformed to the provisions of the Declaration of Helsinki and was approved by the Ethics Committee of Hunan Children's Hospital (no. HCHLL-2022-70, date: August 2, 2022).

**Figure 1:**
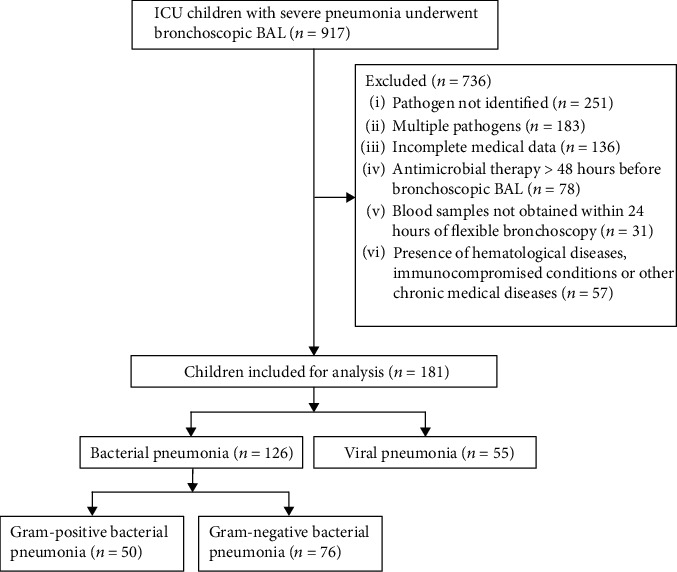
Flowchart of the child selection process for analysis. ICU: intensive care unit; BAL: bronchoalveolar lavage.

Exclusion criteria included pathogens not identified or multiple pathogens (more than one pathogen); incomplete medical data; antimicrobial therapy > 48 hours before bronchoscopic BAL; blood samples not obtained within 24 hours of flexible bronchoscopy; or hematological diseases, immunocompromised conditions, or other chronic medical conditions. A total of 917 children with severe CAP who underwent bronchoscopic BAL were initially enrolled. A total of 736 children were excluded according to the exclusion criteria, and 181 children were finally included in further analysis. The included children were divided into a bacterial pneumonia group and a viral pneumonia group based on the etiological results in BALF. The flowchart of patients in this study is shown in Figure 1.

### 2.2. Data Collection

Flexible bronchoscopy with BAL was routinely performed in response to clinical signs and symptoms. The BAL procedure followed a standardized protocol. Sterile normal saline was instilled through the working channel of a bronchoscope that was placed at the wedged position in a segmental airway. The BALF was aspirated and retrieved using wall suction and collected in sterile containers. BALF was sent to the laboratory department as soon as possible for cytological analysis (differential cell counts including percentages of neutrophils, lymphocytes, alveolar macrophages, and eosinophils); inflammatory biomarker determination (HBP and interleukin-6) and pathogen cultivation, including semiquantitative cultures for bacteria and fungi; PCR for Mycoplasma, Chlamydia, bacteria, and respiratory viruses; and antigen detection for influenza. Bacterial and viral pneumonia were confirmed if the BALF sample had bacterial growth > 10^4^ colony-forming units (CFU)/mL or was bacterial PCR positive and was viral PCR or influenza antigen test positive, respectively. BALF samples for HBP and interleukin-6 detection were centrifuged, and the cell-free supernatant was used for analysis within 1 hour.

Blood samples for inflammatory biomarker measurement were collected within 24 hours of the flexible bronchoscopy procedure and were detected within 2 hours of collection. HBP was examined by a quantitative method of dry immunofluorescence assay (Jet–iStar 3000; JoinStar, Hangzhou, China), and the results were available within 30 minutes. The analytical performance characteristics of HBP were as follows: accuracy for recovery rate ranges from 85.0% to 115.0%, detection sensitivity was 5.90 ng/mL, the linear coefficient of association was above 0.9900 in the range from 5.90 ng/mL to 300.00 ng/mL, the precision for repetitive coefficient of variation was below 10.0%, and for between-run coefficient of variation was below 15.0%. The examination was performed according to the manufacturer's recommendations. Other laboratory tests, including white blood cell (WBC) count, neutrophil percentage (N%), C-reactive protein (CRP), procalcitonin (PCT), and interleukin-6 (IL-6) concentrations, were recorded for further analyses.

To adjust for dilution of BALF and estimate the concentrations of biomarkers in the lung epithelial lining fluid, we determined the urea content in BALF and concomitant blood samples with a quantitative urease assay using an automated analyzer (Beckman Coulter AU5800, USA) according to the procedure provided by the manufacturer. The ratio of blood to BALF urea concentrations was used as a coefficient for dilution to adjust the concentrations of biomarkers, as previously proposed [11].

### 2.3. Statistical Analysis

Measurement data are expressed as medians (interquartile ranges) according to data distribution and were compared using the Mann–Whitney *U* test. Categorical variables are presented as numbers and percentages (%) and were compared using the chi-squared test. Receiver operating characteristic (ROC) curves were established to evaluate the performances of BALF HBP, and IL-6 concentrations and N% and blood HBP, WBC, CRP, PCT, and IL-6 concentrations and N% for predicting bacterial pneumonia. Youden's index was used to determine the optimal cutoff value of the ROC curve. The area under the curve (AUC), sensitivity, and specificity were calculated. The correlation between blood HBP, IL-6, and N% and BALF HBP, IL-6, and N% in the bacterial pneumonia group was analyzed by the Spearman rank correlation test. *P* < 0.05 was regarded as statistically significant. All data were analyzed using SPSS version 19.0 software.

## 3. Results

### 3.1. Patient Characteristics

A total of 181 patients were included in this study, 126 with bacterial pneumonia, and 55 with viral pneumonia. The characteristics of the study population are shown in Table 1. The baseline characteristics of age, ICU hospitalization days and rate of male sex, mechanical ventilation, sepsis and respiratory failure at enrollment, and ICU mortality did not significantly differ between the bacterial pneumonia and viral pneumonia groups. In contrast, blood HBP and PCT concentrations and N% and BALF HBP and IL-6 concentrations and N% were significantly higher in the bacterial pneumonia group than in the viral pneumonia group (*P* < 0.05). However, blood concentrations of IL-6, WBC, and CRP were not significantly different between the two groups (*P* > 0.05).

### 3.2. Pathogens

Pathogens that were identified from BALF in 181 children with severe pneumonia are summarized in Table 2. *Haemophilus influenzae* (*n* = 30) was the most common bacterium, followed by *Streptococcus pneumoniae* (*n* = 27), *Staphylococcus aureus* (*n* = 20), *Klebsiella pneumoniae* (*n* = 15), and *Escherichia coli* (*n* = 11). Respiratory syncytial virus (*n* = 18) was the most common virus, followed by parainfluenza virus (*n* = 11), adenovirus (*n* = 8), Epstein-Barr virus (*n* = 7), and cytomegalovirus (*n* = 5).

### 3.3. Blood and BALF Concentrations of Biomarkers in Different Patient Groups

**Figure 2:**
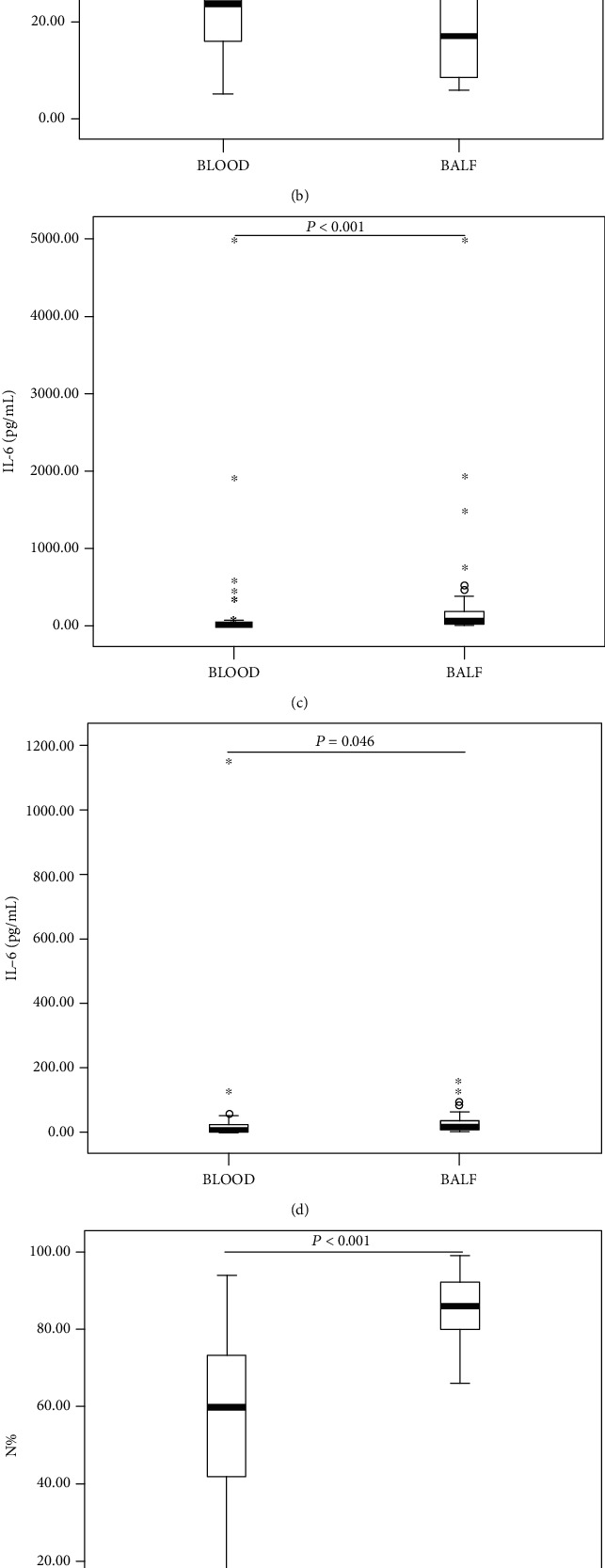
Blood and BALF HBP and IL-6 concentrations and N% in different patient groups. Blood and BALF HBP concentrations in patients with bacterial pneumonia (a) and with viral pneumonia (b), IL-6 concentrations in patients with bacterial pneumonia (c) and with viral pneumonia (d), N% in patients with bacterial pneumonia (e) and with viral pneumonia (f). HBP: heparin-binding protein; IL-6: interleukin-6; N%: neutrophil percentage; BALF: bronchoalveolar lavage fluid. Error bars: 95.00% confidence interval.

Of the 126 children with severe bacterial pneumonia, 50 had gram-positive bacterial infections, and 76 had gram-negative bacterial infections. The concentrations of biomarkers in blood samples (HBP, WBC, N%, CRP, PCT, and IL-6) or in BALF samples (HBP, IL-6, and N%) were not significantly different between the gram-positive bacterial and gram-negative bacterial groups (*P* > 0.05). In the bacterial pneumonia group, HBP and IL-6 concentrations and N% in BALF samples were all significantly higher than those in blood samples (*P* < 0.001). In the viral pneumonia group, the IL-6 concentration in BALF was higher than that in blood (*P* = 0.046); however, HBP concentrations and N% did not significantly differ between the two groups (*P* > 0.05) (Figure 2).

### 3.4. Relationship between Blood and BALF HBP and IL-6 Concentrations and N% in Bacterial Pneumonia Patients

Spearman's rank correlation test revealed that BALF HBP and IL-6 concentrations and N% were lowly correlated with blood HBP and IL-6 concentrations and N% in children with bacterial pneumonia (*r* = 0.439, *P* < 0.001; *r* = 0.250, *P* = 0.005; *r* = 0.235, *P* = 0.008). Blood N% was lowly correlated with the blood HBP concentration (*r* = 0.346, *P* < 0.001). Blood N% was not correlated with the blood IL-6 concentration (*P* > 0.05). BALF N% was positively correlated with BALF HBP and lowly correlated with BALF IL-6 concentrations (*r* = 0.622, *P* < 0.001 vs. *r* = 0.206, *P* = 0.021).

### 3.5. Predictive Value of Blood and BALF Biomarkers for Bacterial Pneumonia

**Figure 3:**
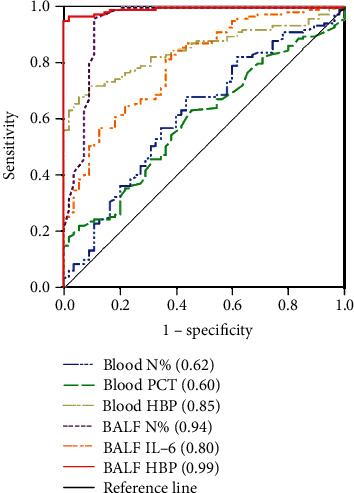
ROC curves for biomarkers in blood and BALF for predicting bacterial pneumonia. HBP: heparin-binding protein; CRP: C-reactive protein; IL-6: interleukin-6; PCT: procalcitonin; WBC: white blood cell count; N%: neutrophil percentage; BALF: bronchoalveolar lavage fluid.

Our univariate analysis revealed that patients with severe bacterial pneumonia had higher blood HBP and PCT concentrations and N% and BALF HBP and IL-6 concentrations and N%. The ability of these biomarkers to predict bacterial pneumonia was assessed using ROC analysis (Figure 3 and Table 3). BALF HBP yielded the greatest predictive power (AUC 0.994), with a sensitivity of 95.24% and specificity of 100.00% at its optimal cutoff value of 74.05 ng/mL, followed by BALF N% (AUC 0.942, with a sensitivity of 96.03% and specificity of 89.09% at its optimal cutoff value of 70.50%) and blood HBP (AUC 0.851, with a sensitivity of 72.22% and specificity of 90.91% at its optimal cutoff value of 50.80 ng/mL).

### 3.6. Comparison of Urea-Adjusted Values for BALF HBP and IL-6 between the Bacterial and Viral Pneumonia Groups

**Figure 4:**
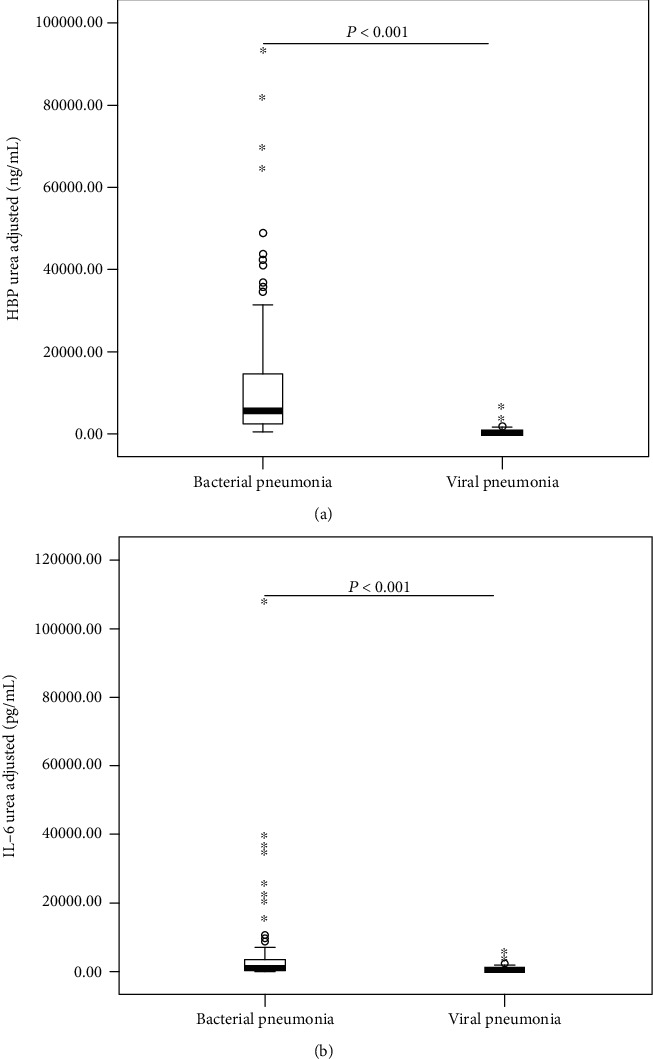
Urea-adjusted values for BALF HBP (a) and IL-6 (b) in children with bacterial or viral pneumonia. HBP: heparin-binding protein; IL-6: interleukin-6; BALF: bronchoalveolar lavage fluid. Error bars: 95.00% confidence interval.

In all 181 children with severe CAP, determination of urea content in BALF and blood samples was performed, allowing estimation of the BALF dilution by the urea method. When adjusting for the dilution factor, children with bacterial pneumonia still had higher concentrations of HBP and IL-6 in BALF than children with viral pneumonia (*P* < 0.001) (Figure 4).

## 4. Discussion

In this study, HBP concentrations in BALF and paired blood samples were determined to predict the etiology of pneumonia in critically ill children with severe CAP. The present study showed that both BALF and blood concentrations of HBP in bacterial pneumonia were higher than those in viral pneumonia. In bacterial pneumonia, the BALF HBP concentration was significantly higher than the blood HBP concentration, and BALF HBP concentration was lowly correlated with the blood HBP concentration. The correlation between BALF HBP concentrations and N% was relatively close to that between blood HBP concentrations and N%. Compared with blood HBP or other biomarkers in BALF and blood, BALF HBP had the best diagnostic performance for predicting bacterial pneumonia. The data indicate that analysis of HBP in BALF is a rapid and useful biomarker for differentiating bacterial pneumonia from viral pneumonia in critically ill children.

BALF, which partially recovers instilled saline and epithelial lining fluid, has been used for both diagnosis and research purposes. Carreto-Binaghi et al. [12] analyzed cytokines and collectins in BALF samples to investigate the pulmonary immunological response of pneumonia patients infected with *Histoplasma capsulatum* and *Pneumocystis jirovecii*. The presence of neutrophils in BALF is considered strongly predictive of a secondary bacterial infection in COVID-19-induced acute respiratory distress syndrome [13]. However, information about the role of HBP in BALF for the differential diagnosis of bacterial pneumonia in critically ill children is scarce. In the current study, the participants were children in the ICU with severe CAP caused by a single pathogen, as determined from BALF samples. The data showed that children with bacterial pneumonia had higher HBP concentrations in BALF. However, the difference in BALF HBP concentrations between gram-positive and gram-negative bacterial pneumonia was not significant. The optimal cutoff value of BALF HBP for predicting bacterial pneumonia was 74.05 ng/mL in the current study, which was much lower than that in a previous study performed by Stjärne Aspelund et al. [7]. In their study, HBP concentrations were assayed by enzyme-linked immunosorbent assay, and the cutoff value of HBP in BALF for predicting pulmonary infection in adult lung transplant recipients was 150 ng/mL. The reasons for this discrepancy may be the differences in study populations, underlying diseases, bacterial species, antibiotic treatment before enrollment, HBP detection method, and other unknown reasons, indicating that the application of HBP in various routine clinical scenarios needs to be investigated.

A previous study revealed that HBP concentrations in BALF in ventilated patients in the ICU were much higher than concentrations in plasma, despite being diluted in the BAL procedure [6]. In the present study, children with bacterial pneumonia had significantly higher HBP concentrations in BALF samples than in blood samples. In the viral pneumonia group, HBP concentrations did not significantly differ between the two samples. In addition, BALF concentrations of HBP were correlated with blood concentrations of HBP in children with bacterial pneumonia. These results are mainly in accordance with a previous study and suggest that it might be possible for HBP to pass the epithelial and endothelial membranes under the condition of increased vascular permeability, which may be caused by an inflammatory response disrupting capillary endothelial and alveolar epithelial barriers, as well as HBP inducing cytoskeletal rearrangement and enhancing the permeability of endothelial cells [14]. This finding indicates that the lungs might be the end organ where neutrophils become activated to release their granular contents and that the lungs might indeed be a source of systemic HBP in critically ill children.

The inflammatory response in the lower respiratory tract regulated by cytokines and other inflammatory mediators is the main immunopathological characteristic of CAP. In response to infection, neutrophils are recruited to the lungs depending on the cytokine signaling pathways of IL-6, nuclear factor kappa B, and tumor necrosis factor. IL-6 in the BALF was reported to be positively correlated with neutrophil counts in the BALF [15]. Other studies have demonstrated that blood HBP and PCT and blood and BALF N% are helpful biomarkers in diagnosing bacterial pneumonia in adult patients [16, 17]. In the present study, blood HBP and PCT concentrations and N% and BALF HBP and IL-6 concentrations and N% were significantly higher in bacterial pneumonia, and BALF N% was lowly correlated with BALF IL-6 concentrations. These results agree with previous studies. In bacterial pneumonia, IL-6 concentrations and N% in BALF samples were both significantly higher than those in blood samples, and BALF IL-6 concentrations and N% were lowly correlated with blood IL-6 concentrations and N%, which indicates that the markers in BALF might mirror systemic biomarkers. Interestingly, the current study showed that BALF N% and HBP concentrations had a closer relationship than blood N% and HBP concentrations. We speculate that the reasons may be that the N% in BALF is higher than that in blood in this study and that the HBP concentration probably reflects the number of activated neutrophils rather than the total neutrophils.

When used to predict bacterial pneumonia, HBP in BALF had the highest AUC value with the best specificity, and N% in BALF had the second highest AUC value with the best sensitivity. These data indicate that the inflammatory biomarkers HBP, PCT, and N% in blood and HBP, IL-6, and N% in BALF are useful for differentiating bacterial pneumonia from viral pneumonia. In these cases, the HBP concentration in BALF seemed to be superior to other biomarkers, and the combination of BALF HBP concentrations and BALF N% may be a better option in routine clinical application.

Given that biomarkers were diluted in the BAL process, we used the urea method as previously described to correct for the BALF dilution factors [11]. The results showed that urea-adjusted values for BALF HBP and IL-6 still had higher concentrations in bacterial pneumonia than in viral pneumonia, which conversely suggests that the direct measurement of biomarkers in BALF samples based on a standardized BAL procedure should be recommended for the sake of convenience and applicability in everyday clinical practice.

This study has several limitations. First, the single center and relatively small sample size of the selected critically ill pediatric population analysis limit the general use of the findings. Second, although it would be a strong indication for bacterial etiology if the concentrations of BALF biomarkers increase even after antimicrobial therapy for 48 hours, the impact of antimicrobial therapy on BALF inflammatory markers remains to be further investigated. Third, cases of mycoplasma pneumonia, mycobacterial pneumonia, fungal pneumonia, and pneumonia caused by other pathogens were not involved, mainly because most children had coinfections and few children had pneumonia caused by only one type of these pathogens. Lastly, PCT concentration in BALF was not measured because of the low expression concentration and little change of PCT in BALF and the detection method being not suitable for the specimen of BALF.

## 5. Conclusions

Our study revealed that BALF HBP is a promising biomarker for strongly predicting bacterial infection in critically ill children with severe CAP and may rapidly and simply provide valuable information for the early differential diagnosis of bacterial pneumonia in routine clinical practice. However, additional multicenter and large-scale studies are required to validate our results.

## Figures and Tables

**Table 1:** Characteristics of the study population.

Characteristic	Bacterial pneumonia (*n* = 126)	Viral pneumonia (*n* = 55)	*P* value
Age, months	9.50 (1.00–192.00)	11.00 (1.00–159.00)	0.790
Male, *n* (%)	93 (73.80)	35 (63.60)	0.167
Mechanical ventilation, *n* (%)	25 (19.8)	10 (18.2)	0.795
Sepsis at enrollment, *n* (%)	6 (4.8)	2 (3.6)	0.735
Respiratory failure at enrollment, *n* (%)	104 (82.5)	45 (81.8)	0.907
ICU hospitalization days	7.00 (5.00–10.00)	8.00 (6.00–10.00)	0.485
ICU mortality, *n* (%)	3 (2.4)	1 (1.8)	0.802
Laboratory data (blood)			
HBP, ng/mL	116.94 (38.97–195.61)	23.81 (15.46–35.53)	<0.001
CRP, mg/L	2.77 (0.50–12.66)	1.14 (0.50–8.00)	0.077
IL-6, pg/mL	10.46 (5.50–24.37)	7.95 (3.12–22.04)	0.061
PCT, ng/mL	0.15 (0.08–0.41)	0.11 (0.06–0.21)	0.035
WBC, 10^9^/L	8.87 (6.31–12.59)	9.28 (6.50–13.04)	0.888
N%	59.70 (41.68–73.23)	47.50 (28.80–64.80)	0.010
Laboratory data (BALF)			
HBP, ng/mL	337.63 (183.81–698.20)	17.07 (8.36–33.84)	<0.001
IL-6, pg/mL	65.76 (24.25–170.20)	13.88 (4.99–34.15)	<0.001
N%	86.00 (79.75–92.00)	38.00 (25.00–64.00)	<0.001

Data were expressed as numbers and percentages (%) for enumeration data or medians (interquartile ranges) for measurement data except for age, which was presented as median and range from minimum to maximum. ICU: intensive care unit; HBP: heparin-binding protein; CRP: C-reactive protein; IL-6: interleukin-6; PCT: procalcitonin; WBC: white blood cell count; N%: neutrophil percentage; BALF: bronchoalveolar lavage fluid.

**Table 2:** Identities of pathogens in children with pneumonia.

Type of pneumonia	Pathogen	Number (percentage)
Bacterial pneumonia (*n* = 126)	Gram-positive bacteria	50 (27.62)
	*Staphylococcus aureus*	20 (11.05)
	*Streptococcus pneumoniae*	27 (14.92)
	Group A streptococcus	3 (1.66)
	Gram-negative bacteria	76 (41.99)
	*Haemophilus influenzae*	30 (16.57)
	*Klebsiella pneumoniae*	15 (8.29)
	*Escherichia coli*	11 (6.08)
	*Enterobacter aerogenes*	2 (1.10)
	*Moraxella catarrhalis*	3 (1.66)
	*Serratia marcescens*	4 (2.21)
	*Enterobacter cloacae*	4 (2.21)
	*Acinetobacter baumannii*	4 (2.21)
	*Aeromonas hydrophila*	1 (0.55)
	*Pseudomonas aeruginosa*	2 (1.10)

Viral pneumonia (*n* = 55)	Respiratory syncytial virus	18 (9.94)
	Parainfluenza virus	11 (6.08)
	Adenovirus	8 (4.42)
	Epstein-Barr virus	7 (3.87)
	Cytomegalovirus	5 (2.76)
	Influenza A	2 (1.10)
	Influenza B	4 (2.21)

**Table 3:** Performance characteristics of biomarkers in blood and BALF for predicting bacterial pneumonia.

Biomarker	Cutoff value	AUC	*P*	95% CI	Sensitivity (%)	Specificity (%)
Blood N%	48.60%	0.621	0.010	0.532–0.710	68.25	56.36
Blood PCT	0.12 ng/mL	0.598	0.035	0.513–0.684	63.49	54.55
Blood HBP	50.80 ng/mL	0.851	<0.001	0.797–0.905	72.22	90.91
BALF N%	70.50%	0.942	<0.001	0.896–0.988	96.03	89.09
BALF IL-6	19.26 pg/mL	0.800	<0.001	0.732–0.867	81.75	63.64
BALF HBP	74.05 ng/mL	0.994	<0.001	0.987–1.001	95.24	100.00

HBP: heparin-binding protein; IL-6: interleukin-6; PCT: procalcitonin; N%: neutrophil percentage; BALF: bronchoalveolar lavage fluid; AUC: area under the curve; 95% CI: 95% confidence interval.

## Data Availability

The data used to support the findings of this study are available from the corresponding author upon request.
